# Environmental Factors Predicting Blood Lead Levels in Pregnant Women in the UK: The ALSPAC Study

**DOI:** 10.1371/journal.pone.0072371

**Published:** 2013-09-05

**Authors:** Caroline M. Taylor, Jean Golding, Joseph Hibbeln, Alan M. Emond

**Affiliations:** 1 Centre for Child and Adolescent Health, School of Social and Community Medicine, University of Bristol, Bristol, United Kingdom; 2 NIAAA, National Institutes of Health, Rockville, Maryland, United States of America; University of Cincinnati, United States of America

## Abstract

**Background:**

Lead is a widespread environmental toxin. The behaviour and academic performance of children can be adversely affected even at low blood lead levels (BLL) of 5–10 µg/dl. An important contribution to the infant's lead load is provided by maternal transfer during pregnancy.

**Objectives:**

Our aim was to determine BLL in a large cohort of pregnant women in the UK and to identify the factors that contribute to BLL in pregnant women.

**Methods:**

Pregnant women resident in the Avon area of the UK were enrolled in the Avon Longitudinal Study of Parents and Children (ALSPAC) in 1991–1992. Whole blood samples were collected at median gestational age of 11 weeks and analysed by inductively coupled plasma dynamic reaction cell mass spectrometry (n = 4285). Self-completion postal questionnaires were used to collect data during pregnancy on lifestyle, diet and other environmental exposures. Statistical analysis was carried out with SPSS v19.

**Results:**

The mean±SD BLL was 3.67±1.47 (median 3.41, range 0.41–19.14) µg/dl. Higher educational qualification was found to be one of the strongest independent predictor of BLL in an adjusted backwards stepwise logistic regression to predict maternal BLL <5 or ≥5 µg/dl (odds ratio 1.26, 95% confidence interval 1.12–1.42; p<0.001). Other predictive factors included cigarette smoking, alcohol and coffee drinking, and heating the home with a coal fire, with some evidence for iron and calcium intake having protective effects.

**Conclusion:**

The mean BLL in this group of pregnant women is higher than has been found in similar populations in developed countries. The finding that high education attainment was independently associated with higher BLL was unexpected and currently unexplained. Reduction in maternal lead levels can best be undertaken by reducing intake of the social drugs cigarettes, alcohol and caffeine, although further investigation of the effect of calcium on lead levels is needed.

## Introduction

Lead is a neurotoxic metal that is widespread in the environment. The removal of lead from water pipes, paint, and food cans, and the introduction of lead-free petrol in most countries has reduced exposure to lead in recent years. Geographical areas of high levels of exposure, including those with smelters, lead shot works, or battery manufacture and recycling remain a concern. Although atmospheric lead levels have declined in the UK, food and drink, water, dust and soil remain important sources of exposure [1].

Once absorbed from the gastrointestinal tract or the respiratory system, lead is transported bound to erythrocytes and accumulates in bone (with a half-life of approximately 30 years). Pregnancy is a critical time for exposure to lead for the mother and fetus. An increased demand for calcium means that maternal turnover of bone increases during this time [2], and bone becomes the main source of lead in the blood, especially if dietary lead is low [3]. Lead crosses the placenta freely, so the fetus is exposed to lead in utero, with the ratio of fetal:maternal blood lead being about 0.7–0.9 [4]–[6].

Infants and children are particularly at risk of lead exposure. Not only are they born with lead transferred to them from their mothers, but they have a greater exposure to lead from dust and soil than adults [7]. In addition, their gastrointestinal absorption of lead is greater than in adults (about 50% vs 10–15%, respectively [8]), and they are more vulnerable to the effects of lead on the developing nervous system [9]. Low-level exposure to lead remains a serious and largely undetected problem in children: blood lead levels (BLL) below that accepted internationally as the level of concern [10] have been shown to be harmful to the academic performance and behaviour of children [11].

The effects of fetal lead exposure make it important to identify factors that contribute to maternal BLL, especially those that could be modified to minimise transfer to the fetus. However, there may also be direct adverse effects on the mother even at relatively low levels, notably to blood pressure [12]. Hypertension in pregnancy has at least two distinct adverse effects: (1) it may predispose women to the development of future cardiovascular disease and increase the severity of the disease; (2) it can lead to pre-eclampsia, which is associated with substantial maternal morbidity and mortality. There are multiple factors that have been identified as being probable contributors to maternal BLL. Maternal levels are greatest where there is a clear exposure to environmental contamination, for example living in a mining area or area of high pollution, or near a lead smelting works or solid waste incinerator [13]–[16]. Consumption of marine meat and blubber from areas with environmental contamination [17] or wild birds and game containing lead shot [18] are also associated with high maternal levels. Other factors identified as having a positive association with maternal BLL include coffee and alcohol consumption, smoking (both active and passive), age, gestational age, weight gain during pregnancy, higher haemoglobin and haematocrit levels, ethnicity, lower social class, lower socioeconomic status, lower educational attainment, lower family income and seafood consumption [6], [15], [19]–[24]. Calcium intake, taking iron and folic acid supplements, increased parity and low physical activity seem to protect against increased lead levels in pregnancy [21], [23], [25].

Our primary aim was to model associations of BLL in pregnancy with social, environmental and other factors in a large observational cohort study in the UK. BLL in pregnancy in the UK have only been reported four times previously in the UK, most recently in 1996 from data collected in 1991 on a small sample (n = 183) [26]. A secondary aim was to identify an association between maternal BLL and child BLL. By studying a cohort of pregnant women enrolled in the Avon Longitudinal Study of Parents and Children (ALSPAC), we had an opportunity to include a greater number of women than has ever been reported on before and to provide new data on BLL in women during pregnancy in the UK.

## Methods

### The ALSPAC study

The study sample was derived from the ALSPAC study, a population-based study investigating environmental and genetic influences on the health, behaviour and development of children. All pregnant women in the former Avon Health Authority with an expected delivery date between 1 April 1991 and 31 December 1992 were eligible for the study; 14,541 pregnant women were initially enrolled, resulting in a cohort of 14,062 live births [27]. The social and demographic characteristics of this cohort were similar to those found in UK national census surveys [28]. Further details of ALSPAC are available at www.bris.ac.uk/alspac.

### Collection, storage and analysis of blood samples

#### Maternal blood samples

Whole blood samples were collected in acid-washed vacutainers (Becton and Dickinson, Oxford, UK) by midwives as early as possible in pregnancy. The median gestational age at the time of blood sampling was 11 weeks (range 1–42 weeks, interquartile range 9–13 weeks). Whole blood samples were stored in the original tube at 4°C at the collection site before being transferred to the central Bristol laboratory within 1–4 days. Samples were at ambient temperature during transfer (up to 3 h). They were then stored at 4°C until analysis. Samples were analysed for lead using inductively-coupled plasma mass spectrometry in standard mode (R. Jones; Centers for Disease Control (CDC), Bethesda, MD, USA; CDC Method 3009.1; for details see Text S1). The analyses were completed on 4285 women for Pb. One sample had a Pb level below the limit of detection (0.29 µg/dl): this sample was assigned a value of 0.7 times the lower limit of detection.

#### Child blood samples

Details of the selection of the subsample of children and analysis of the blood samples have been reported in detail by Golding et al. [29] and Chandramouli et al. [11] In brief, a 10% randomly selected sample of parents whose babies were born in the last 6 months of the ALSPAC study were invited to attend a research clinic (Children in Focus, CIF). At age 30 months, parental consent was sought for a venous blood sample, and was given by 81% of the 1135 children in the CIF group. The sample was drawn into lead-free tubes from 653 (71%) of children attending the clinic. However, 69 samples were insufficient, leaving 582 samples for analysis. Analysis was by atomic absorption spectrometry (Southampton General Hospital, UK) with appropriate quality controls.

### Questionnaires

The mothers received four postal self-completion questionnaires during pregnancy. The questionnaires are available from the study website (http://www.bristol.ac.uk/alspac/researchers/resources-available/data-details/questionnaires/).

Information on environmental and lifestyle factors included data on age, parity, social class, highest educational qualification, housing type, fuel used to heat the home, cigarette smoking history and exposure to passive smoking (partner or other household member smoked), and alcohol, coffee and tea consumption. If questions were repeated in different questionnaires (e.g. alcohol consumption, cigarette smoking, coffee consumption), then the responses from the earliest questionnaire were used for analysis in order to correspond with the timing of the blood sampling.

The haemoglobin level was extracted from the obstetric clinic records (the first recorded level was used in order to correspond with the gestational time of the blood sample for lead analysis). The Neighbourhood Quality Index (score 1–12) was calculated from a composite index of scores for ‘Lively’ ‘Friendly’, ‘Noisy’, ‘Clean’, ‘Attractive’ and ‘Polluted/dirty’ neighbourhood (a higher score indicates a higher quality neighbourhood). Total daily calcium and iron intakes were derived from food frequency questionnaires sent to the women at 32 weeks gestation. Information on taking iron and calcium supplements in the previous 3 months was extracted from a questionnaire at 32 weeks gestation.

### Identification of possible predictors of BLL and statistical analysis

Factors that had a possible association with BLL were identified from the literature: linear correlation was then used to identify factors that had an association with BLL. To reduce the risk of over-adjusting the models and substantially reducing the number of cases included, not all significant variables were included in the models (e.g. maternal ethnic group as sample numbers in the non-white groups were small). All factors with p≥0.05 were rejected (taking iron supplements was included in the models, but taking calcium supplements was not included as there was no linear association with BLL; see Table S1 for a list of variables that were considered). The effects of all types of alcohol (wine, beer, sprits and other types of alcohol) were combined into one variable (alcohol); both types of coffee (caffeinated and decaffeinated) were combined into one variable (coffee). Separate models were used to identify the relative effects of different types of alcohol and of different types of coffee. Initial analyses were performed with and without the use of a wood fire as a confounder: no statistical differences were apparent so wood fires were not included in the final analyses.

Statistical analysis was done with SPSS version 19 (IBM Corp.). Values are shown as mean±SD. The characteristics of the sample of pregnant women included in the study were compared with those of the rest of the ALSPAC cohort (chi-square test). The demographics of the study population were analysed with BLL as a continuous variable and as a categorical variable (<5 and ≥5 µg/dl) (ANOVA and chi-square test, respectively).

Hierarchical backwards stepwise logistic regression was used to examine the effects of the predictors with BLL as a categorical variable (gestational age and haemoglobin were entered in Block 1 and the remaining variables in Block 2). Categorical outcomes were recoded as integers with equal increments for this analysis. Variables were removed from the models if F ≥0.100. The effect of transforming BLL to a log_e_ scale was examined in an identical model but with log_e_ BLL as the dependent variable.

Regression diagnostics were used to check that the models fitted the observed data well and to identify any cases that had undue influence on the model. No cases were identified as outliers for the linear or the logistic regression model. The variance inflation factor and tolerance statistic for each variable remaining in the model, as well as the correlation matrix, were used to check for collinearlity. There was no evidence for any collinearity (maximum variance inflation factor 1.112 (‘Hb at first clinic visit’), minimum tolerance statistic 0.873 (‘number of cigarettes per day at present’), maximum correlation –0.030 (‘Hb at first clinic visit’ vs ‘Taking Fe this pregnancy’).

Data on BLL in 233 mother–child pairs were available. The correlation of maternal and child BLL was examined by Pearson's r test (two tailed).

### Ethics statement

Ethics approval for the study was obtained from the ALPAC Ethics and Law Committee and Local Research Ethics Committees. Informed written consent was obtained for analysis of biological samples and linking results to the data collected from questionnaires.

## Results

### Characteristics of the sample of pregnant women with BLL data

The subsample had a slightly higher educational attainment (p = 0.004) and tended to be slightly older (p<0.001), but there were no other statistically significant differences (see Table S2 for characteristics of the subsample of pregnant women with BLL compared with the rest of the ALSPAC cohort).

### Maternal BLL

**Figure 1:**
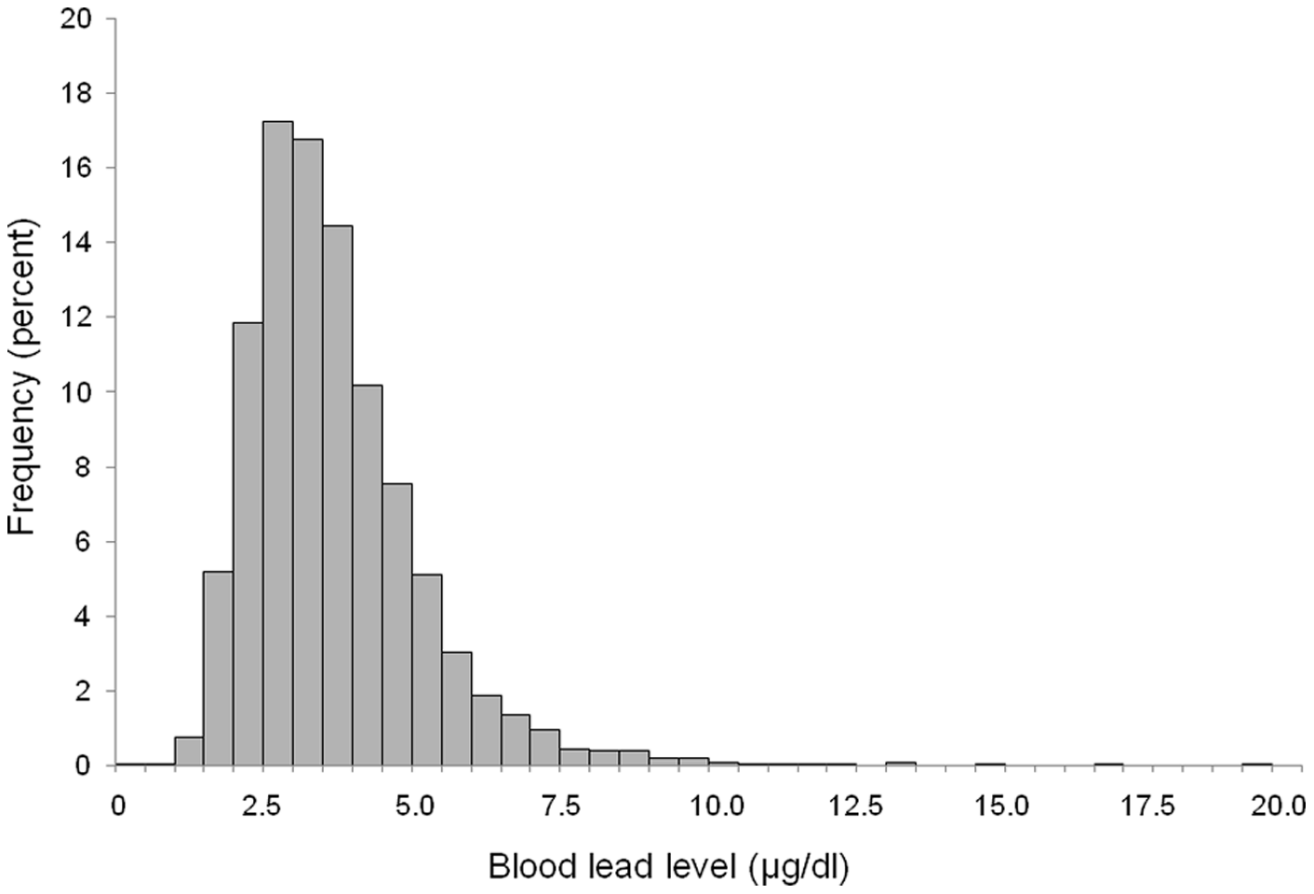
Frequency distribution of blood lead levels in pregnant women (n = 4285).

The mean maternal BLL was 3.67±1.47 µg/dl (n = 4285; geometric mean 3.43, median 3.42, range 0.41–19.14 µg/dl). The distribution was slightly skewed (Fig. 1). The BLL was ≥5 µg/dl in 619 women (14.4%) and ≥10 µg/dl in 15 women (0.4%). Women with the highest educational qualification (degree) had higher BLL than those without a degree (4.07±1.68 (n = 566) vs 3.59±1.69 (n = 3021), ANOVA p<0.001) (see Table 1). Women who had lived in Avon all their lives had lower BLL than those who had not (3.59±1.55 vs 3.73±1.38 µg/dl; p = 0.002). Women of Indian, Pakistani and Bangladeshi ethnicity had significantly higher BLL than White women (4.37±1.92 (n = 23) vs 3.65±1.47 (n = 3585), respectively, ANOVA p = 0.019). There was no significant difference in the BLL in women who were White vs Black (3.84±1.40 (n = 42), ANOVA p = 0.394) or White vs Other ethnicities (3.82±1.03 (n = 22), ANOVA p = 0.600).

**Table 1:** Maternal BLL by highest maternal educational qualification.

	n	Mean±SD (µg/dl)	Range (µg/dl)
Degree	566	4.07±1.69	1.34–13.28
A Level	841	3.69±1.48	1.05–14.70
O Level	1226	3.54±1.47	0.41–19.14
Vocational	345	3.44±1.17	1.36–10.30
None/CSE	709	3.62±1.34	0.78–9.46

A, advanced; CSE, Certificate of School Education; O, ordinary.

Women with BLL ≥5 µg/dl were older (p<0.001), had higher educational attainment (p<0.001), higher social class (p<0.001) and lower parity (p = 0.013), and were more likely to smoke (p<0.001) and consume alcohol (p<0.001) (see Table S3).

### Predictors of BLL as a continuous variable

The models using BLL or log_e_ BLL were broadly similar. The results using BLL are presented for ease of interpretation (for comparison, the results using log_e_ BLL are shown in Table S4). The overall model predicted 10.9% of the variance in BLL levels (Table 2). Highest educational qualification, alcohol consumption, cigarette smoking, coffee consumption (caffeinated and decaffeinated combined, using a coal fire as a source of heating and haemoglobin level (all p<0.001) were all strongly and independently associated with increased BLL. Lower BLL was associated with a higher Neighbourhood Quality Index, increasing parity, and increasing gestational age when the sample was taken (p = 0.021, p<0.001 and p = 0.017, respectively). Dietary calcium intake and taking iron supplements were protective against high lead levels (p = 0.001 and p = 0.046, respectively). Alcohol, smoking cigarettes, haemoglobin level and highest educational attainment made the greatest relative contributions to BLL (relative *R* ^2^ 17.6%, 16.5%, 14.3% and 11.0%, respectively).

**Table 2:** Regression coefficients for variables that remained after backward stepwise elimination procedures in a multiple linear regression model predicting maternal BLL.

Predictor variable	Unstandardised coefficient: B (SE) (×100)	Standardised coefficient: β (×100)	t	*p* value	Relative *R* ^2^ (%)
Age (years)	2.8 (0.7)	8.8	3.816	<0.001	7.7
Gestational age (weeks)	–1.6 (0.7)	–4.8	–2.383	0.017	3.3
Parity (n)	–12.4 (3.7)	–7.2	–3.316	<0.001	1.1
Haemoglobin (g/dl)	14.3 (3.6)	8.5	4.013	<0.001	14.3
Taken Fe supplements during this pregnancy (yes)	–16.4 (8.2)	–4.2	–1.994	0.046	2.2
Dietary Ca intake (g/day)	–36.2 (10.9)	–6.7	–3.321	0.001	2.2
Neighbourhood quality index^a^	–3.2 (1.4)	–4.8	–2.317	0.021	3.3
Highest maternal educational attainment^b^	13.7 (2.7)	11.4	5.034	<0.001	11.0
Alcohol (units/week)	5.9 (0.8)	14.8	7.177	<0.001	17.6
Cigarettes (n/day)	4.2 (0.7)	13.4	6.181	<0.001	16.5
Coffee^c^ (cups/day)	1.0 (0.3)	7.9	3.801	<0.001	6.6
Coal fire (yes)	39.2 (9.9)	8.2	3.971	<0.001	7.7
No. of dogs	8.6 (4.9)	3.6	1.737	0.083	6.6

Overall *R* ^2^ = 10.9%; p<0.001.

Model was hierarchical with gestational age and haemoglobin entered in Block 1 and remaining variables in Block 2.

Removed from the model (F≥0.100): social class, dietary iron intake, always lived in Avon.

a The Neighbourhood Quality Index (score 1–12) was calculated from a composite index of scores for ‘Lively’ ‘Friendly’, ‘Noisy’, ‘Clean’, ‘Attractive’ and ‘Polluted/dirty’ neighbourhood (a higher score indicates a higher quality neighbourhood).

b Reference: Certificate of Secondary Education (CSE)/no qualifications.

c Caffeinated plus decaffeinated.

### Predictors of BLL as a categorical variable

The strongest statistically significant categorical predictor of BLL was having a coal fire (p = 0.008), followed by highest education attainment (p<0.001), haemoglobin level (p = 0.014), alcohol consumption (p = 0.001), age (p = 0.006), cigarette smoking (p = 0.001) and coffee consumption (p<0.001) (Table 3). Dietary calcium intake was protective against high BLL (p = 0.041).

**Table 3:** Regression coefficients for potential variables that remained after backward stepwise logistic regression to predict maternal BLL <5 or ≥5 µg/dl.

Predictor variable	Unstandardised coefficient B (SE) (× 100)	OR	95% CI	*p* value
Age (years)	4.3 (1.5)	1.043	1.013–1.076	0.006
Haemoglobin (g/dl)	17.9 (7.3)	1.196	1.037–1.380	0.014
Parity (n)	–13.9 (8.1)	0.871	0.743–1.020	0.086
Dietary calcium intake (mg/day)	–48.6 (23.7)	0.615	0.386–0.980	0.041
Highest maternal educational attainment^a^	23.1 (5.9)	1.260	1.123–1.415	<0.001
Alcohol (units/week)	4.7 (1.4)	1.048	1.019–1.078	0.001
Cigarettes (n/day)	4.0 (1.2)	1.040	1.015–1.066	0.001
Coffee (cups/day)^b^	1.8 (0.5)	1.019	1.009–1.028	<0.001
Coal fire (yes)	47.0 (17.7)	1.600	1.130–2.266	0.008

Model overall p<0.001.

Model was hierarchical with gestational age and haemoglobin entered in Block 1 and remaining variables in Block 2.

Removed from the model (F≥0.100): gestational age, month of blood sample, dietary iron intake, taking iron supplements, social class, Neighbourhood Quality Index, number of dogs, always lived in Avon.

a Reference: Certificate of Secondary Education (CSE)/no qualifications.

b Caffeinated plus decaffeinated.

### Smoking, alcohol consumption, and coffee and tea consumption

The number of units of alcohol consumed per week was correlated with BLL (r = 0.059, p<0.001). Wine consumption was the strongest statistically significant predictor of BLL (p<0.001), followed in turn by beer (p = 0.003) and spirits (p = 0.020) (Table S5).

Caffeinated coffee was a statistically significantly stronger predictor of BLL than decaffeinated coffee (p<0.001) when the model was adjusted for tea consumption (Table S6).

### Child BLL and correlation with maternal BLL

As previously reported [11], [29], the mean child BLL at 30 months was 4.22±3.12 (median 3.32, range 0.83–27.56; n = 582) µg/dl. In the 233 mother–child pairs the maternal BLL was 3.53±1.51 (median 3.29, range 1.22–13.48) µg/dl and the child BLL at 30 months was 4.21±3.25 (median 3.31, range 0.83–26.63) µg/dl. There was a significant correlation between maternal and child BLL (r = 0.278, n = 233; p<0.001).

## Discussion

The mean BLL of 3.67±1.47 (geometric mean 3.43) µg/dl is higher than has been found in similar groups of pregnant women. In studies published from 2000 onwards where no specific source of environmental exposure was identified by the authors and the women were not resident in major cities in developing countries where there are likely to be high levels of pollution, BLL in pregnancy have ranged from a mean value of 1.8±1.63 µg/dl (n = 211; USA) [6] to 2.1 µg/dl (n = 160, Canada) [30] (Table 4). With regard to the UK, the most recent study to our knowledge reporting BLL in pregnancy is that of Watt et al. (1996) [26], who found levels of 3.65 µg/dl (Glasgow; geometric mean) in a small group of women (n = 138) in 1991. By comparison, a more recent UK-wide study found geometric mean levels of 2.7±2.1 and 2.6±0.05 µg/dl in women of childbearing age aged 16–24 (n = 321) and 25–45 years (n = 1276), respectively, in a cohort that was studied in 1995 [31]. These data suggest a decline in blood leads in women in the UK with time, but more data are needed to confirm this.

**Table 4:** Comparison of BLL in pregnancy.

Authors	Country	Blood lead (µg/dl)	n	Year of survey	Sampling time
Osman et al. (2000) [44]	Sweden (Stockholm)	1.139^a^	88	1994–1999	36 weeks gestation
Smargiassi et al. (2002) [30]	Canada (Montreal)	2.1^b^	160	–	–
Sowers et al. (2002) [45]	USA (New Jersey)	1.2±0.03^c^	705	–	Once in each trimester
Schell et al. (2003) [6]	USA (Albany, NY)	1.9±1.68 1.8±1.63 1.8±1.65	211	1992–1998	1st trimester 2nd trimester 3rd trimester
Harville et al. (2005) [46]	USA (Pittsburgh, IL)	1.93 (0.55–4.70)	140	1992–1995	At delivery
Abdelouahab et al. (2010) [47]	France (Nancy and Poitiers)	1.84±1.21	160	–	24–28 weeks gestation
Gundacker et al. (2010) [48]	Austria (Vienna)	1.04–8.4 (2.5^a^)	52	2005	Week 34–38
Hansen et al. (2011) [49]	Norway	0.82±0.04 (0.22–4.11)	210	2007–2009	Second trimester
Sanders et al. (2012) [50]	Sweden	0.89^b^ (0.19–7.72)	211	2009–2011	Third trimester
Present study (2013)	UK (Bristol area)	3.67±1.47 (3.43^b^)	4285	1991–1992	Median 11 weeks gestation

Studies shown are those published from 2000 onwards only where there was no specific source of environmental lead exposure and the participants were not resident in major cities in developing countries (developing/developed countries were defined according to the International Monetary Fund definition [51]).

For studies reporting BLL during pregnancy in which the author identified a specific source of environmental lead exposure and/or participants lived in a major city in a developing country, see Table S7.

a Median; ^b^geometric mean; ^c^standard error; values in parentheses are ranges.

There are several factors that should be taken in account when comparing maternal BLL between studies (see Table 4). First, the sample sizes in published studies range widely from 18 to 1577. 27 Second, several analytical techniques for lead analysis have been used, including commercial kits, atomic absorption spectroscopy and inductively coupled plasma mass spectroscopy. Third, there is a range of gestational ages at which the blood samples have been taken. Fourth, the date that the study was performed is not always reported.

Considering these points, the present study has several strengths. First, it has a large number of participants (n = 4285) with good socio-demographic background data. Second, we used inductively-coupled plasma mass spectrometry, with appropriate quality controls for the maternal BLL: this is considered to be one of the most accurate and precise analytical methods currently available. BLL are thought to follow a U-shaped curve during gestation [21], so that a comparison of levels taken near the beginning and end of pregnancy is valid: most studies that report on the timing of the blood sample have taken blood in the third trimester or at about the time of birth (Table 4). Comparison of previous results with the present results taken in the first trimester is therefore appropriate. Finally, our blood samples were collected in 1991–1992: although this is earlier than for some studies published recently, the results still represent an important contribution to the literature due to the lack of comparable data in the UK and the large sample size.

It is possible that local environmental conditions may have contributed to the relatively high BLL in our sample as there is a legacy of lead mining and industrial processing in the Avon area. Being African-American has been found to be associated with high BLL in studies in the USA [6], [21], but we did not find any effect of being Afro-Caribbean in our study. The relatively small number of Afro-Caribbean women in our study may account for this, as may differences in the demographics of Afro-Caribbean people in the UK compared with African-American people in the USA. We did find that Asian people had significantly higher levels than White women, although again the sample size was small. An association between being Asian and higher BLL in pregnancy has not been noted specifically before, but cultural practices common in Asian populations, such as the application of henna to the hair and hands and kohl to the eyes, have been shown to be associated with higher BLL [24].

The multiple linear regression model of predictors of BLL explained about 11% of the variability in blood lead: the remainder is be likely to be accounted for by water, diet, air, dust, in utero transmission to the mother, and exposure through occupation and hobbies. Recent studies have found higher levels of the variability explained by their models: for example, the model of Al-Saleh et al [24], who also used a backwards stepwise multiple regression model that contained similar predictors to our model, explained about 37% of the variability. This difference may reflect the likelihood that environmental factors to which our population were exposed were not accounted for in the model.

It was surprising that high educational attainment was a relatively strong predictor of BLL, since low socioeconomic status, low social class and low education attainment are usually associated with high BLL in pregnancy [19], [21], [22], [24]. A possible explanation is that women with higher educational attainment in our sample lived in older housing stock rather than on modern estates, and were perhaps involved in the renovation of older properties: this might cause exposure to lead-based paints, through sanding old woodwork, and to large amounts of dust that has accumulated over many decades (as mentioned earlier, the Avon area of the UK, which includes the city of Bristol, has a rich history of lead-mining and industrial processing from Roman times up to the recent past). We did not have any data on the ages of the houses that our participants lived in, so are not able to verify this. Social class, however, did not emerge as a significant predictor of BLL once educational attainment was taken into account. Social class categories were derived from current or previous employment: women at this stage of life may be ‘underemployed’ because of childcare commitments, and so this variable may not be a true reflection of their social status. The assumption that more disadvantaged populations have higher levels of exposure to environmental pollutants has recently been questioned [32], and our results support this view.

An effect of having a coal fire as a source of domestic heating on BLL during pregnancy has not been reported before, but air-borne particulates from coal-burning are known to contain several heavy metals, including lead [33]. Coal-burning is also known to generate polycyclic aromatic hydrocarbons, which can cause adverse birth outcomes and developmental delay [34]. There was a weak positive effect of owning dogs in both models: an association with dog-owning was noted in a subsample of the children from the women in the present study [29] that is likely to have been caused by dogs bringing lead-containing soil and dust into the home.

Alcohol consumption and cigarette smoking both made significant independent contributions to BLL in pregnancy, as has been found previously [15], [23], [24], [35], [36]. Tobacco is known to have high lead levels as the plant takes up lead from the soil, and this is reflected in the smoke that cigarettes produce [37]. This represents an important prenatal exposure to lead for newborn infants. Several studies have reported that alcohol is a significant predictor of BLL, and the effect of wine in particular has also been noted [15]. Wine and beer may contain high lead levels, possibly due to contamination from fittings used in manufacturing and processing at that time in the UK in the case of beer, and from degradation of the lead capsules on wine bottles [38]. In addition, it has been suggested that alcohol ingestion, particularly if unaccompanied by food, increases the relative absorption of lead [39].

The UK Food Standards Agency has recently calculated that the food group ‘Beverages’ makes the largest single contribution of lead to the UK diet (17%) [40]. There are two mechanisms whereby coffee could contribute to blood levels. First, coffee is known to contain high levels of lead [1]. Second, caffeine may lead to a small negative calcium balance through a weak interference with calcium absorption, leading to an increase in bone resorption, although this effect is thought to be of importance only in those with low calcium intake [41]. We did not find any independent effect of tea, which also contains caffeine, although less than is present than in coffee.

Lead is transported in blood bound to haemoglobin, so it was unsurprising that haemoglobin level was found to be a predictor of BLL in both models. The median gestational age that the blood sample was taken was 11 weeks, so it is unlikely that there was any effect of haemodilution at this stage of pregnancy. As pregnancy progresses, the effect of haemodilution is counterbalanced by the increase in lead released into the blood through increased bone turnover, resulting in the U-shaped curve observed by Hertz-Piccioto et al. [21]. The rate of bone turnover is thought to be greatest in the third trimester [21] and so this may also account for the independent effect of gestational age.

We identified several factors that were protective against high BLL. The protective effect of increasing parity has been noted before [22], [23], contradicting Rothenburg et al. who speculated that changes in the structure of the placenta with each pregnancy would alter the blood flow and enable a greater transfer of lead with increasing parity [42]. Calcium, iron and lead are thought to compete for gastrointestinal binding sites, and a high dietary calcium intake could reduce rate of bone turnover: protective effects of calcium (and milk products) and iron have been found in several other studies [19], [21], [23], [25]. Calcium supplementation (1200 mg/day) during pregnancy in a study with initial BLL similar to that in the present study has been shown to decrease BLL [43]. It was not possible to calculate overall calcium and iron intakes in the present study as the level of supplementation taken was not quantified.

We have shown a statistically significant correlation between maternal BLL during pregnancy and child BLL at age 30 months. Effects of early childhood lead exposure on the academic performance and behaviour of these children at age 7–8 years has already been reported [11]. As the ratio of fetal:maternal blood lead is about 0.8 [4]–[6], the infants would have been born with a mean level of about 2.8 µg/dl, with further lead exposure during early infancy accounting for a further approximately 1.4 µg/dl. In this group, therefore, maternal transfer of lead in utero is likely to have made a substantial contribution to the infant's BLL, which in turn was associated with adverse educational and behavioural outcomes.

The limitations of our study were: (1) the bias in the sample (women who had BLL measurement were older and had higher educational levels than the rest of the ALSPAC cohort); (2) not including data on environmental exposures in the models that might account for the remainder of the variability in BLL. It is of note that although the independent effects of maternal education, smoking, alcohol consumption and coffee consumption were statistically significant, the odds ratios are relatively modest. With regard to the child's BLL, the samples were not analysed with the same method or at the same time as maternal BLL, but both methods included rigorous quality control procedures that enable confidence in comparison of the results.

## Conclusion

Lead exposure is harmful to children even at low levels. We identified higher educational attainment as an unexpected independent predictor of BLL in our cohort of pregnant women. This may be reflected from exposure to lead during renovation work in older properties: if this is the case then pregnant women should be advised to avoid household maintenance activities in older properties, especially if the household uses coal for domestic heating. Cigarette smoking, alcohol intake and coffee consumption were significant modifiable predictors of maternal lead levels during pregnancy. These factors will cause increased transfer of lead to the fetus. Our findings also reinforce existing public health messages on the harmful effects of cigarette smoking and alcohol consumption during pregnancy, and suggest that these should be extended to reducing the consumption of coffee during pregnancy. Increased calcium and iron intakes may reduce lead levels in pregnancy, but this needs further exploration in carefully designed trials.

## Supporting Information

Text S1 **Details of analysis of samples for lead.**
(DOCX)

Table S1 **List of variables associated with blood lead levels that were considered for inclusion in regression analyses (Pearson's r (two-tailed), ANOVA or t test).**
(DOCX)

Table S2 **Characteristics of sample of mothers with data on blood lead levels.**
(DOCX)

Table S3 **Demographics of study population.**
(DOCX)

Table S4 **Regression coefficients for variables that remained after backward stepwise elimination procedures in a multiple linear regression model predicting maternal log_e_ blood lead level.**
(DOCX)

Table S5 **Regression coefficients for type of alcohol in a backwards linear regression model.**
(DOCX)

Table S6 **Regression coefficients for type of coffee in a backwards linear regression model.**
(DOCX)

Table S7 **Comparison of blood lead levels in pregnancy from studies in which the authors identified a specific source of environmental lead exposure and/or participants were resident in a major city in a developing country.**
(DOCX)

## References

[1] European Food Safety Authority Panel on Contaminants in the Food Chain (2010) Scientific opinion on lead in food. EFSA J 8: 1570–1717.

[2] Gulson BL, Jameson CW, Mahaffey KR, Mizon KJ, Korsch MJ, et al (1997) Pregnancy increases mobilization of lead from maternal skeleton. J Lab Clin Med 130: 51–62. 9242366 10.1016/s0022-2143(97)90058-5

[3] Gulson BL, Mahaffey KR, Jameson CW, Patison N, Law AJ, et al (1999) Impact of diet on lead in blood and urine in female adults and relevance to mobilization of lead from bone stores. Environ Health Perspect 107: 257–263. 10090703 10.1289/ehp.99107257 PMC1566515

[4] Amaral JH, Rezende VB, Quintana SM, Gerlach RF, Barbosa F Jr, et al (2010) The relationship between blood and serum lead levels in peripartum women and their respective umbilical cords. Basic Clin Pharmacol Toxicol 107: 971–975. 20629654 10.1111/j.1742-7843.2010.00616.x

[5] Rudge CV, Rollin HB, Nogueira CM, Thomassen Y, Rudge MC, et al (2009) The placenta as a barrier for toxic and essential elements in paired maternal and cord blood samples of South African delivering women. J Environ Monitor 11: 1322–1330. 10.1039/b903805a 20449220

[6] Schell LM, Denham M, Stark AD, Gomez M, Ravenscroft J, et al (2003) Maternal blood lead concentration, diet during pregnancy, and anthropometry predict neonatal blood lead in a socioeconomically disadvantaged population. Environ Health Perspect 111: 195–200. 12573905 10.1289/ehp.5592 PMC1241350

[7] McMichael AJ, Baghurst PA, Robertson EF, Vimpani GV, Wigg NR (1985) The Port Pirie cohort study. Blood lead concentrations in early childhood. Med J Aust 143: 499–503. 4069047

[8] Ziegler EE, Edwards BB, Jensen RL, Mahaffey KR, Fomon SJ (1978) Absorption and retention of lead by infants. Pediatr Res 12: 29–34. 643372 10.1203/00006450-197801000-00008

[9] Bellinger DC (2004) Lead. Pediatrics 113: 1016–1022. 15060194

[10] Centers for Disease Control and Prevention (2005) Preventing lead poisoning in young children. Atlanta, GA. Available: http://www.cdc.gov/nceh/lead/publications/prevleadpoisoning.pdf. Accessed 15 July 2013.

[11] Chandramouli K, Steer C, Ellis M, Emond A (2009) Effects of early childhood lead exposure on academic performance and behaviour of school age children. Archives of Disease in Childhood 94: 844–848. 19770197 10.1136/adc.2008.149955

[12] Wells EM, Navas-Acien A, Herbstman JB, Apelberg BJ, Silbergeld EK, et al (2011) Low-level lead exposure and elevations in blood pressure during pregnancy. Environ Health Perspect 119: 664–669. 21292600 10.1289/ehp.1002666 PMC3094418

[13] Baghurst PA, Robertson EF, McMichael AJ, Vimpani GV, Wigg NR, et al (1987) The Port Pirie Cohort Study: lead effects on pregnancy outcome and early childhood development. Neurotoxicology 8: 395–401. 2443882

[14] Eik Anda E, Nieboer E, Dudarev AA, Sandanger TM, Odland JO (2007) Intra- and intercompartmental associations between levels of organochlorines in maternal plasma, cord plasma and breast milk, and lead and cadmium in whole blood, for indigenous peoples of Chukotka, Russia. J Environ Monit 9: 884–893. 17671671 10.1039/b706717h

[15] Lagerkvist BJ, Ekesrydh S, Englyst V, Nordberg GF, Soderberg HA, et al (1996) Increased blood lead and decreased calcium levels during pregnancy: A prospective study of Swedish women living near a smelter. Am J Public Health 86: 1247–1252. 8806376 10.2105/ajph.86.9.1247 PMC1380587

[16] Reis MF, Sampaio C, Brantes A, Aniceto P, Melim M, et al (2007) Human exposure to heavy metals in the vicinity of Portuguese solid waste incinerators–Part 2: biomonitoring of lead in maternal and umbilical cord blood. Int J Hyg Environ Health 210: 447–454. 17347042 10.1016/j.ijheh.2007.01.020

[17] Bjerregaard P, Hansen JC (2000) Organochlorines and heavy metals in pregnant women from the Disko Bay area in Greenland. Sci Total Environ 245: 195–202. 10682367 10.1016/s0048-9697(99)00444-1

[18] Butler Walker J, Houseman J, Seddon L, McMullan E, Tofflemire K, et al (2006) Maternal and umbikical cord blood levels of mercury, lead, cadmium, and essential trace elements in Arctic Canada. Enviro Res 100: 295–319. 10.1016/j.envres.2005.05.006 16081062

[19] Baghurst PA, McMichael AJ, Vimpani GV, Robertson EF, Clark PD, et al (1987) Determinants of blood lead concentrations of pregnant women living in Port Pirie and surrounding areas. Med J Aust 146: 69–73. 3796424 10.5694/j.1326-5377.1987.tb136265.x

[20] Grandjean P, Weihe P, Jorgensen PJ, Clarkson T, Cernichiari E, et al (1992) Impact of maternal seafood diet on fetal exposure to mercury, selenium, and lead. Arch Environ Health 47: 185–195. 1596101 10.1080/00039896.1992.9938348

[21] Hertz-Picciotto I, Schramm M, Watt-Morse M, Chantala K, Anderson J, et al (2000) Patterns and determinants of blood lead during pregnancy. Am J Epidemiol 152: 829–837. 11085394 10.1093/aje/152.9.829

[22] Llop S, Aguinagalde X, Vioque J, Ibarluzea J, Guxens M, et al (2011) Prenatal exposure to lead in Spain: cord blood levels and associated factors. Sci Total Environ 409: 2298–2305. 21397928 10.1016/j.scitotenv.2011.02.004

[23] Al-Jawadi AA, Al-Mola ZW, Al-Jomard RA (2009) Determinants of maternal and umbilical blood lead levels: a cross-sectional study, Mosul, Iraq. BMC Res Notes 2: 47. 19309527 10.1186/1756-0500-2-47 PMC2663773

[24] Al-Saleh I, Shinwari N, Mashhour A, Mohamed Gel D, Rabah A (2011) Heavy metals (lead, cadmium and mercury) in maternal, cord blood and placenta of healthy women. Int J Hyg Environ Health 214: 79–101. 21093366 10.1016/j.ijheh.2010.10.001

[25] Rothenberg SJ, Karchmer S, Schnaas L, Perroni E, Zea F, et al (1994) Changes in serial blood lead levels during pregnancy. Environ Health Perspect 102: 876–880. 9644197 10.1289/ehp.94102876 PMC1567359

[26] Watt GC, Britton A, Gilmour WH, Moore MR, Murray GD, et al (1996) Is lead in tap water still a public health problem? An observational study in Glasgow. BMJ 313: 979–981. 8892418 10.1136/bmj.313.7063.979 PMC2352296

[27] Boyd A, Golding J, Macleod J, Lawlor DA, Fraser A, et al (2013) Cohort profile: The 'Children of the 90s'–the index offspring of the Avon Longitudinal Study of Parents and Children. Int J Epidemiol 42: 111–127. 22507743 10.1093/ije/dys064 PMC3600618

[28] Fraser A, Macdonald-Wallis C, Tilling K, Boyd A, Golding J, et al (2013) Cohort profile: The Avon Longitudinal Study of Parents and Children: ALSPAC mothers cohort. Int J Epidemiol 42: 97–110. 22507742 10.1093/ije/dys066 PMC3600619

[29] Golding J, Smith M, Delves HT, Taylor H, Team AS (1998) The ALSPAC study on lead in children. Norwich: Insitute for Environment and Health. 35–39. Available: http://www.cranfield.ac.uk/health/researchareas/environmenthealth/ieh/ieh%20publications/r9.pdf. Accessed 15 July 2013.

[30] Smargiassi A, Takser L, Masse A, Sergerie M, Mergler D, et al (2002) A comparative study of manganese and lead levels in human umbilical cords and maternal blood from two urban centers exposed to different gasoline additives. Sci Total Environ 290: 157–164. 12083707 10.1016/s0048-9697(01)01071-3

[31] Primatesta P, Dong W, Bost L, Poulter NR, Delves HT (1998) Survey of blood lead levels in the population in England, 1995. Norwich, UK: Medical Research Council, Institute for Environmental Health. 9–34. Available: http://www.cranfield.ac.uk/health/researchareas/environmenthealth/ieh/ieh%20publications/r9.pdf. Accessed 15 July 2013.

[32] Vrijheid M, Martinez D, Aguilera I, Ballester F, Basterrechea M, et al (2012) Socioeconomic status and exposure to multiple environmental pollutants during pregnancy: evidence for environmental inequity? J Epidemiol Commun Health 66: 106–113. 10.1136/jech.2010.117408 20974841

[33] Gromov S, Ginzburg V (1998) Estimation of heavy metal emission from coal-fired power plants in Russia. Trans Ecol Environ 21: 597–606.

[34] Perera FP, Li Z, Whyatt R, Hoepner L, Wang S, et al (2009) Prenatal airborne polycyclic aromatic hydrocarbon exposure and child IQ at age 5 years. Pediatrics 124: e195–202. 19620194 10.1542/peds.2008-3506 PMC2864932

[35] Grandjean P, Olsen NB, Hollnagel H (1981) Influence of smoking and alcohol consumption on blood lead levels. Int Arch Occup Environ Health 48: 391–397. 7298208 10.1007/BF00378687

[36] Hertz-Picciotto I (2000) The evidence that lead increases the risk for spontaneous abortion. Am J Ind Med 38: 300–309. 10940968 10.1002/1097-0274(200009)38:3<300::aid-ajim9>3.0.co;2-c

[37] Bernhard D, Rossmann A, Wick G (2005) Metals in cigarette smoke. IUBMB Life 57: 805–809. 16393783 10.1080/15216540500459667

[38] Smart GA, Pickford CJ, Sherlock JC (1990) Lead in alcoholic beverages: a second survey. Food Addit Contam 7: 93–99. 2307272 10.1080/02652039009373825

[39] Newton D, Pickford CJ, Chamberlain AC, Sherlock JC, Hislop JS (1992) Elevation of lead in human blood from its controlled ingestion in beer. Hum Exp Toxicol 11: 3–9. 1354457 10.1177/096032719201100101

[40] Food Standards Agency (2009) Measurement of the concentrations of metals and other elements from the 2006 UK total diet study. Available: http://www.food.gov.uk/multimedia/pdfs/fsis0109metals.pdf. Accessed 15 July 2013.

[41] Heaney RP (2002) Effects of caffeine on bone and the calcium economy. Food Chem Toxicol 40: 1263–1270. 12204390 10.1016/s0278-6915(02)00094-7

[42] Rothenberg SJ, Schnaas L, Cansinoortiz S, Perronihernandez E, Delatorre P, et al (1989) Neuro-behavioral deficits after low-level lead exposure in neonates–the Mexico City Pilot Study. Neurotoxicol Teratol 11: 85–93. 2733657 10.1016/0892-0362(89)90046-9

[43] Ettinger AS, Lamadrid-Figueroa H, Tellez-Rojo MM, Mercado-Garcia A, Peterson KE, et al (2009) Effect of calcium supplementation on blood lead levels in pregnancy: a randomized placebo-controlled trial. Environ Health Perspect 117: 26–31. 19165383 10.1289/ehp.11868 PMC2627861

[44] Osman K, Akesson A, Berglund M, Bremme K, Schutz A, et al (2000) Toxic and essential elements in placentas of Swedish women. Clin Biochem 33: 131–138. 10751591 10.1016/s0009-9120(00)00052-7

[45] Sowers M, Jannausch M, Scholl T, Li W, Kemp FW, et al (2002) Blood lead concentrations and pregnancy outcomes. Arch Environ Health 57: 489–495. 12641194 10.1080/00039890209601442

[46] Harville EW, Hertz-Picciotto I, Schramm M, Watt-Morse M, Chantala K, et al (2005) Factors influencing the difference between maternal and cord blood lead. Occup Environ Med 62: 263–269. 15778260 10.1136/oem.2003.012492 PMC1740989

[47] Abdelouahab N, Huel G, Suvorov A, Foliguet B, Goua V, et al (2010) Monoamine oxidase activity in placenta in relation to manganese, cadmium, lead, and mercury at delivery. Neurotoxicol Teratol 32: 256–261. 19744554 10.1016/j.ntt.2009.08.010

[48] Gundacker C, Frohlich S, Graf-Rohrmeister K, Eibenberger B, Jessenig V, et al (2010) Perinatal lead and mercury exposure in Austria. Sci Total Environ 408: 5744–5749. 20825977 10.1016/j.scitotenv.2010.07.079

[49] Hansen S, Nieboer E, Sandanger TM, Wilsgaard T, Thomassen Y, et al (2011) Changes in maternal blood concentrations of selected essential and toxic elements during and after pregnancy. J Environ Monit 13: 2143–2152. 21738945 10.1039/c1em10051c

[50] Sanders AP, Flood K, Chiang S, Herring AH, Wolf L, et al (2012) Towards prenatal biomonitoring in North Carolina: assessing arsenic, cadmium, mercury, and lead levels in pregnant women. PLoS One 7: e31354. 22427803 10.1371/journal.pone.0031354 PMC3302877

[51] World Economic and Financial Surveys (2012) World Economic Outlook. Growth Resuming, Dangers Remain. Washington DC: International Monetary Fund. Available: http://www.imf.org/external/pubs/ft/weo/2012/01/pdf/text.pdf. Accessed 15 July 2013.

