# Supplementary material for: Environmental Factors Predicting Blood Lead Levels in Pregnant Women in the UK: The ALSPAC Study
Source: PLoS One. 2013 Sep 5;8(9):e72371. doi: 10.1371/journal.pone.0072371 (PMC3764234; doi:10.1371/journal.pone.0072371)
Supplement: Text S1 — Details of analysis of samples for lead. (DOCX) [file pone.0072371.s001.docx]

**Text S1**Details of analysis of samples for lead

Clotted whole blood was quantitatively transferred to a digestion tube using concentrated nitric acid. Nitric acid was used to solubilise and stabilize metal in solution and to aid the complete transfer of the blood matrix out of the original blood vial. The mass of blood transferred from the original tube was determined by difference weighing on an analytical balance. The volume of blood transferred for digestion was determined by calculation using an assumed density derived from the literature (average of range) and the mass of blood transferred. The blood sample, in a nitric acid and hydrogen peroxide mix, was heated in a microwave at a controlled temperature and time during which the organic matrix of the blood was decomposed, removing clots. A spike of rhodium was added to the blood digestion prior to heating to allow for correction for any evaporative loss during the heating process. The resulting digestate was further diluted 1+9 to reduce the concentration of the acid and total dissolves solids in the digestion matrix. ICP-DRC-MS internal standards Ir and Te were at a constant concentration in all blanks, calibrators and samples (added at the time of the 1+9 dilution of digestate).
