# Supplementary material for: Environmental Factors Predicting Blood Lead Levels in Pregnant Women in the UK: The ALSPAC Study
Source: PLoS One. 2013 Sep 5;8(9):e72371. doi: 10.1371/journal.pone.0072371 (PMC3764234; doi:10.1371/journal.pone.0072371)
Supplement: Table S1 — List of variables associated with blood lead levels that were considered for inclusion in regression analyses (Pearson's r (two-tailed), ANOVA or t test). (DOCX) [file pone.0072371.s002.docx]

**Table S1** List of variables associated with blood lead levels that were considered for inclusion in regression analyses (Pearson’s r (two-tailed), ANOVA or t test)

| **Significant variable p<0.05** | **Non-significant variable (p≥0.05)** |
| --- | --- |
| Mother’s age ^a^ | BMI |
| Parity ^a^ | Pre-pregnancy weight |
| Gestational age at time of sample^a^ | Height |
| Hb at first measurement if <18 weeks^a^ | Breast-fed last baby/duration of breast-feeding |
| Mother’s ethnic group | Partner’s ethnic group |
| Month of year of blood sample |  |
|  |  |
| Maternal social class^a^ | Paternal social class |
| Maternal highest educational qualification^a^ |  |
| Paternal highest educational qualification |  |
|  |  |
| Always lived in Avon ^a^ |  |
| Where parents lived at time of birth |  |
|  |  |
| Crowding index | Refurbishment (number of areas painted, papered or re-carpeted in previous year) |
| Neighbourhood quality index^a^ |  |
|  |  |
| Housing type | Home ownership status |
| Lowest level of accommodation |  |
|  |  |
| Domestic heating: wood fire, coal fire^a^ | Domestic heating: central heating, paraffin, calor gas, mains gas |
|  |  |
|  |  |
| Number of dogs^a^ | Any pets, number of cats |
|  |  |
| Cups of coffee^a^, cups of decaffeinated coffee^a^ | Cups of tea, cups of decaffeinated tea, cans of cola, cans of decaffeinated cola |
| Cups of herbal tea |  |
| Alcohol^a^: beer/lager, wine, spirits, other alcohol |  |
|  |  |
| Smoking^a^: number of cigarettes at present, ever smoked, age started smoking, smoking 3 months before pregnancy, tar intake, nicotine intake | Passive smoking: passive (partner or other household member) |
|  |  |
| Smoked cannabis in the 6 months pre-pregnancy and in the last 3 months |  |
|  |  |
| Calcium intake at 32 weeks^a^ |  |
| Iron intake at 32 weeks^a^ |  |
| Iron supplements in last 3 months^a^ | Calcium supplements in last 3 months |
| Being vegetarian at present | Being vegan at present |
|  |  |
| CAM: homeopathic product use, herbal product use | Use of other CAM products |

CAM, complementary and alternative medicines.

^a^Included in models. To reduce the risk of over-adjusting the models, not all significant variables were included in the models (e.g. maternal ethnic group as sample numbers in the non-white group were small).
