# Supplementary material for: Environmental Factors Predicting Blood Lead Levels in Pregnant Women in the UK: The ALSPAC Study
Source: PLoS One. 2013 Sep 5;8(9):e72371. doi: 10.1371/journal.pone.0072371 (PMC3764234; doi:10.1371/journal.pone.0072371)
Supplement: Table S2 — Characteristics of sample of mothers with data on blood lead levels. (DOCX) [file pone.0072371.s003.docx]

**Table S2** Characteristics of sample of mothers with data on blood lead levels

| **Variable** | **Mothers with blood lead level value, n (%)** | **Rest of ALSPAC cohort, n (%)** | ***p* value**  **(chi square test)** |
| --- | --- | --- | --- |
| n | 4285 (29.5) | 10257 (70.5) |  |
| Age (years) |  |  |  |
| <20 | 240 (6.1) | 628 (6.5) | 0.002 |
| ≥20–24 | 719 (18.2) | 1935 (20.2) |  |
| ≥25–29 | 1537 (38.9) | 3799 (39.6) |  |
| ≥30–34 | 1105 (28.0) | 2389 (24.9) |  |
| ≥35 | 346 (8.8) | 847 (8.8) |  |
| Maternal education |  |  |  |
| None/CSE | 709 (19.2) | 1785 (20.6) | <0.001 |
| Vocational | 345 (9.4) | 870 (10.1) |  |
| O level | 1226 (33.3) | 3047 (35.2) |  |
| A level | 841 (22.8) | 1930 (22.3) |  |
| Degree | 566 (15.4) | 1021 (11.8) |  |
| Spent whole life in Avon |  |  | 0.132 (NS) |
| No | 1859 (47.6) | 4379 (46.2) |  |
| Yes | 2044 (52.4) | 5099 (53.8) |  |
| Maternal social class |  |  |  |
| I | 200 (6.6) | 391 (5.6) | 0.383 (NS) |
| II | 960 (31.7) | 2182 (31.3) |  |
| III (non-manual) | 1276 (42.2) | 2998 (43.0) |  |
| III (manual) | 228 (7.5) | 555 (8.0) |  |
| IV | 360 (9.7) | 843 (9.9) |  |
| V | 67 (2.2) | 153 (2.2) |  |
| Ethnicity |  |  | 0.131 (NS) |
| White | 3585 (97.6) | 8342 (97.3) |  |
| Black (African, Caribbean, other) | 42 (1.1) | 87 (1.0) |  |
| Indian, Pakistani, Bangladeshi | 23 (0.6) | 60 (0.7) |  |
| Other | 22 (0.6) | 87 (1.0) |  |
| Housing |  |  |  |
| Mortgaged/owned | 2827 (72.7) | 6930 (73.4) | 0.426 (NS) |
| Rented/other | 1062 (27.3) | 2516 (26.6) |  |
| Maternal smoking in pregnancy |  |  |  |
| Yes | 985 (25.3) | 2276 (24.6) | 0.397 (NS) |
| No | 2905 (74.7) | 6968 (75.4) |  |
| Paternal smoking in pregnancy |  |  |  |
| Yes | 1411 (37.9) | 3321 (37.5) | 0.641 (NS) |
| No | 2310 (62.1) | 5540 (62.5) |  |
| Parity |  |  |  |
| 0 | 1684 (44.5) | 4085 (45.4) | 0.276 (NS) |
| 1 | 1303 (34.4) | 3132 (34.8) |  |
| 2 | 554 (14.6) | 1261 (14.0) |  |
| 3 | 176 (4.7) | 350 (3.9) |  |
| >3 | 67 (1.8) | 162 (1.9) |  |
