# Supplementary material for: Environmental Factors Predicting Blood Lead Levels in Pregnant Women in the UK: The ALSPAC Study
Source: PLoS One. 2013 Sep 5;8(9):e72371. doi: 10.1371/journal.pone.0072371 (PMC3764234; doi:10.1371/journal.pone.0072371)
Supplement: Table S3 — Demographics of study population. (DOCX) [file pone.0072371.s004.docx]

**Table S3**Demographics of study population

| **Variable** | **Continuous** | | | **Categorical** | | |
| --- | --- | --- | --- | --- | --- | --- |
|  | **n (%)** | **Blood lead level (mean±SD) (µg/dl)** | **p value for**  **effect of variable on blood lead level (ANOVA)** | **Blood lead level  <5 µg/dl** | **Blood lead level  ≥5 µg/dl** | **p value for difference between categories (chi square)** |
|  |  |  |  | n (%) | n (%) |  |
| n | 4285 |  |  | 3665 (85.5) | 619 (14.5) |  |
| Age (years) |  |  |  |  |  |  |
| <20 | 240 (6.1) | 3.75±1.40 | <0.001 | 196 (5.8) | 44 (7.9) | <0.001 |
| ≥20–24 | 719 (18.2) | 3.48±1.16 |  | 656 (19.4) | 63 (11.3) |  |
| ≥25–29 | 1537 (38.9) | 3.57±1.42 |  | 1334 (39.4) | 203 (36.3) |  |
| ≥30–34 | 1105 (28.0) | 3.73±1.55 |  | 933 (27.5) | 172 (30.7) |  |
| ≥35 | 346 (8.9) | 4.14±1.83 |  | 268 (7.9) | 78 (13.9) |  |
| Maternal education |  |  | <0.001 |  |  | <0.001 |
| None/CSE | 709 (19.2) | 3.62±1.34 |  | 619 (19.6) | 90 (17.0) |  |
| Vocational | 345 (9.4) | 3.44±1.17 |  | 308 (9.7) | 37 (7.0) |  |
| O level | 1226 (33.3) | 3.54±1.47 |  | 1074 (34.0) | 152 (28.8) |  |
| A level | 841 (22.8) | 3.69±1.48 |  | 725 (23.0) | 116 (22.0) |  |
| Degree | 566 (15.4) | 4.07±1.69 |  | 433 (13.7) | 133 (25.2) |  |
| Spent whole life in Avon |  |  | 0.002 |  |  | 0.001 |
| Yes | 2044 (52.4) | 3.59±1.55 |  | 1789 (53.4) | 255 (46.0) |  |
| No | 1859 (47.6) | 3.73±1.38 |  | 1560 (46.6) | 299 (54.0) |  |
| Maternal social class |  |  | <0.001 |  |  | <0.001 |
| I | 200 (6.6) | 4.06±1.87 |  | 162 (6.3) | 38 (8.7) |  |
| II | 960 (31.7) | 3.86±1.57 |  | 784 (30.3) | 176 (40.5) |  |
| III non-manual | 1276 (42.2) | 3.49±1.34 |  | 1125 (43.5) | 151 (34.7) |  |
| III manual | 228 (7.5) | 3.62±1.37 |  | 200 (7.7) | 28 (6.4) |  |
| IV | 293 (9.7) | 3.60±1.46 |  | 263 (10.2) | 30 (6.9) |  |
| V | 67 (2.2) | 3.74±1.55 |  | 55 (2.1) | 12 (2.8) |  |
| Ethnicity |  |  | 0.092 (NS) |  |  | 0.001 |
| White | 3585 (97.6) | 3.65±1.47 |  | 3086 (97.9) | 499 (96.0) |  |
| Black (African, Caribbean, other) | 42 (0.01) | 3.84±1.40 |  | 34 (1.1) | 8 (1.5) |  |
| Indian, Pakistani, Bangladeshi | 23 (0.01) | 4.37±1.92 |  | 13 (0.4) | 10 (1.9) |  |
| Other | 22 (0.01) | 3.81±1.03 |  | 19 (0.6) | 3 (0.6) |  |
| Housing |  |  | 0.553 (NS) |  |  | 0.060 (NS) |
| Mortgaged/owned | 2857 (72.7) | 3.67±1.49 |  | 2406 (85.1) | 421 (87.5) |  |
| Rented/other | 1062 (27.3) | 3.64±1.41 |  | 929 (14.9) | 133 (12.5) |  |
| Alcohol (measures per week) |  |  |  |  |  |  |
| 0 | 1856 (67.6) | 3.47±1.36 | <0.001 | 1649 (69.3) | 207 (56.6) | <0.001 |
| 1–9 | 780 (28.4) | 3.85±1.46 |  | 657 (27.6) | 123 (33.6) |  |
| 10–19 | 87 (3.2) | 4.61±2.10 |  | 59 (2.5) | 28 (7.7) |  |
| ≥20 | 22 (0.8) | 4.59±1.94 |  | 14 (0.6) | 9 (2.2) |  |
| Maternal smoking (n per day) |  |  |  |  |  |  |
| 0 | 2905 (74.7) | 3.55±1.39 | <0.001 | 2546 (76.5) | 359 (64.6) | <0.001 |
| 1–9 | 406 (10.4) | 4.06±1.77 |  | 321 (9.6) | 85 (15.3) |  |
| 10–19 | 427 (11.0) | 3.92±1.45 |  | 342 (10.3) | 85 (15.3) |  |
| 20–29 | 146 (3.8) | 4.05±1.59 |  | 120 (3.6) | 26 (4.7) |  |
| ≥30 | 6 (0.2) | 4.47±1.02 |  | 5 (0.1) | 1 (0.2) |  |
| Paternal smoking in pregnancy |  |  | 0.052 (NS) |  |  | 0.847 (NS) |
| Yes | 1411 (37.9) | 3.72±1.44 |  | 1207 (37.8) | 204 (38.5) |  |
| No | 2310 (62.1) | 3.62±1.47 |  | 1984 (62.2) | 326 (61.5) |  |
| Parity |  |  | <0.001 |  |  | 0.021 |
| 0 | 1683 (44.4) | 3.80±1.55 |  | 1410 (43.5) | 273 (50.4) |  |
| 1 | 1303 (34.4) | 3.50±1.32 |  | 1138 (35.1) | 165 (30.4) |  |
| 2 | 554 (14.6) | 3.55±1.38 |  | 487 (15.0) | 67 (12.4) |  |
| 3 | 176 (4.7) | 3.81±1.76 |  | 152 (4.7) | 24 (4.4) |  |
| >3 | 67 (1.8) | 3.90±1.53 |  | 54 (1.7) | 113 (2.4) |  |

CSE, Certificate of Secondary Education.
