# Supplementary material for: Environmental Factors Predicting Blood Lead Levels in Pregnant Women in the UK: The ALSPAC Study
Source: PLoS One. 2013 Sep 5;8(9):e72371. doi: 10.1371/journal.pone.0072371 (PMC3764234; doi:10.1371/journal.pone.0072371)
Supplement: Table S4 — Regression coefficients for variables that remained after backward stepwise elimination procedures in a multiple linear regression model predicting maternal loge blood lead level. (DOCX) [file pone.0072371.s005.docx]

**Table S4**Regression coefficients for variables that remained after backward stepwise elimination procedures in a multiple linear regression model predicting maternal log_e_ blood lead level

| **Predictor variable** | **Unstandardised coefficient: B (SE) (×100)** | **Standardised coefficient: β (×100)** | **t** | ***p* value** | **Relative *R*^2^ (%)** |
| --- | --- | --- | --- | --- | --- |
| Age (years) | 0.6 (0.3) | 7.5 | 3.260 | 0.001 | 6.8 |
| Gestational age (weeks) | –0.4 (0.2) | –4.7 | –2.325 | 0.020 | 4.1 |
| Parity (n) | –3.4 (0.9) | –7.8 | –3.588 | <0.001 | 1.4 |
| Haemoglobin (g/dl) | 3.6 (0.9) | 8.5 | 4.008 | <0.001 | 10.8 |
| Taken Fe supplements during this pregnancy (yes) | –5.2 (2.1) | –5.3 | –2.507 | 0.012 | 2.7 |
| Dietary Ca intake (g/day) | –7.3 (2.8) | –5.4 | –2.647 | 0.002 | 1.4 |
| Neighbourhood quality index^a^ | –0.9 (0.4) | –5.3 | –2.551 | 0.011 | 4.1 |
| Highest maternal educational attainment^b^ | 3.3 (0.7) | 10.7 | 4.745 | <0.001 | 10.8 |
| Alcohol (units/week) | 13.0 (0.2) | 13.0 | 6.056 | <0.001 | 14.9 |
| Cigarettes (n/day) | 1.0 (0.2) | 13.1 | 6.034 | <0.001 | 18.9 |
| Coffee^c^ (cups/day) | 0.3 (0.1) | 8.1 | 3.920 | <0.001 | 9.5 |
| Coal fire (yes) | 11.7 (2.5) | 9.6 | 4.682 | <0.001 | 12.1 |
| No. of dogs | 2.8 (1.2) | 4.6 | 2.255 | 0.024 | 2.7 |

Model was hierarchical with gestational age and haemoglobin entered in Block 1 and remaining variables in Block 2.

Overall *R*^2^=10.5%; *p*<0.001.

Removed from the model (F≥0.100): social class, dietary iron intake, always lived in Avon.

^a^The Neighbourhood Quality Index (score 1–12) was calculated from a composite index of scores for ‘Lively’ ‘Friendly’, ‘Noisy’, ‘Clean’, ‘Attractive’ and ‘Polluted/dirty’ neighbourhood (a higher score indicates a higher quality neighbourhood).

^b^Reference: Certificate of Secondary Education (CSE)/no qualifications.

^c^Caffeinated plus decaffeinated.
