# Supplementary material for: Environmental Factors Predicting Blood Lead Levels in Pregnant Women in the UK: The ALSPAC Study
Source: PLoS One. 2013 Sep 5;8(9):e72371. doi: 10.1371/journal.pone.0072371 (PMC3764234; doi:10.1371/journal.pone.0072371)
Supplement: Table S5 — Regression coefficients for type of alcohol in a backwards linear regression model. (DOCX) [file pone.0072371.s006.docx]

**Table S5**Regression coefficients for type of alcohol in a backwards linear regression model

| **Predictor variable** | **Unstandardised coefficient: B (SE)** | **Standardised coefficient: β** | **t** | ***p* value** |
| --- | --- | --- | --- | --- |
| Lager/beers (half pints per week) | 0.003 (0.001) | 0.050 | 3.017 | 0.003 |
| Wines (glasses per week) | 0.010 (0.001) | 0.135 | 8.164 | <0.001 |
| Spirits (no. of measures per week) | 0.004 (0.002) | 0.039 | 2.321 | 0.020 |

*R*^2^=2.7%; *p*<0.001.

Adjusted for Other types of alcohol.
