# Supplementary material for: Environmental Factors Predicting Blood Lead Levels in Pregnant Women in the UK: The ALSPAC Study
Source: PLoS One. 2013 Sep 5;8(9):e72371. doi: 10.1371/journal.pone.0072371 (PMC3764234; doi:10.1371/journal.pone.0072371)
Supplement: Table S6 — Regression coefficients for type of coffee in a backwards linear regression model. (DOCX) [file pone.0072371.s007.docx]

**Table S6** Regression coefficients for type of coffee in a backwards linear regression model

| **Predictor variable** | **Unstandardised coefficient: B (SE)** | **Standardised coefficient: β** | **t** | ***p* value** |
| --- | --- | --- | --- | --- |
| Coffee (cups per day) | 0.002 (0.000) | 0.113 | 6.895 | <0.001 |
| Decaffeinated coffee (cups per day) | 0.001 (0.000) | 0.038 | 2.341 | 0.019 |

*R*^2^=1.3%; *p*<0.001.

Adjusted for tea, decaffeinated tea
