# Supplementary material for: Environmental Factors Predicting Blood Lead Levels in Pregnant Women in the UK: The ALSPAC Study
Source: PLoS One. 2013 Sep 5;8(9):e72371. doi: 10.1371/journal.pone.0072371 (PMC3764234; doi:10.1371/journal.pone.0072371)
Supplement: Table S7 — Comparison of blood lead levels in pregnancy from studies in which the authors identified a specific source of environmental lead exposure and/or participants were resident in a major city in a developing country. (DOCX) [file pone.0072371.s008.docx]

**Table S7** Comparison of blood lead levels in pregnancy from studies in which the authors identified a specific source of environmental lead exposure and/or participants were resident in a major city in a developing country

| **Authors** | **Country** | **Blood lead (µg/dl)** | **n** | **Year of survey** | **Comment** |
| --- | --- | --- | --- | --- | --- |
| Bjerregaard and Hansen (2000) [[](#_ENREF_1)1] | Greenland (Disko Bay) | 3.57±4.15 | 180 | 1994–1996 | High consumption of marine meat and blubber |
| Raghunath et al. (2000) [[](#_ENREF_2)2] | India (Mumbai) | 6.4±1.69^b^ | 148 | 1993–1997 | Major city in developing country |
| Durska (2001) [[3](#_ENREF_3)] | Poland (Szczecin) | 1.33 | 83 | – |  |
| Ataniyazova et al. (2001) [[](#_ENREF_4)4] | Uzbekistan (Karakalpakstan) | 6.15^a^ | 18 | 2005–2006 | Area of high pollution |
| Sanin et al. (2001) [[](#_ENREF_5)5] | Mexico (Mexico City) | 9.7±4.1 | 329 | 1994–1995 | High environmental exposure |
| Smargassi et al. (2002) [[](#_ENREF_6)6] | France (Paris) | 5.4±1.4^b^ | 206 | – | High petrol lead exposure |
| Hanning et al. (2003) [[](#_ENREF_7)7] | Canada (Northern Ontario) | 2.07±0.003 | 70 |  | High wildfowl consumption |
| Yao and Huang (2003) [[](#_ENREF_8)8] | China (Beijing) | 4.34 | 1151 | – | Area of high pollution |
| Rahman and Hakeem (2003) [[](#_ENREF_9)9] | Pakistan (Karachi) | 9.91±4.44 (2.28–36.35 ) | 74 | – | Environmental pollution |
| Wang et al. (2004) [[1](#_ENREF_10)0] | China | 7.4 (3.52–24.6)^b^  6.59 (2.0–17.2)  5.43 (2.79–18.7) | 89 |  | Rural  Industrial  Urban |
| Kirel et al. 2005 [[11](#_ENREF_11)] | Turkey (Eskisehir) | 2.8±1.5 | 143 |  | Urban area |
| Butler Walker et al. (2006) [[1](#_ENREF_12)2] | Canada (Arctic) | 3.36 (0.207–17.8) | 385 | 1995–1999 | High game and wildbird consumption |
| Vigeh et al. (2006) [[13](#_ENREF_13)] | Iran (Tehran) | 5.09±2.01 (range 1.9-10.1)  4.82±2.22 (1.7–24.6) | 31  365 | 2003-2004 | After delivery |
| Kawata et al. (2006) [[1](#_ENREF_14)4] | China (Yunnnan Province) | 6.73±2.96 | 100 | – | Area of high pollution |
| Lamadrid-Figueroa et al. (2006) [[1](#_ENREF_15)5] | Mexico (Mexico City) | 6.975±4.658  6.263±4.069  6.902±4.466 | 203  222  194 | 1997–1999 | High environmental exposure |
| Eik Anda et al. (2007) [[1](#_ENREF_16)6] | Russia (Chukotka) | 5.0±3.3 | 48 | 2001–2002 | Area of high pollution |
| Reis et al. (2007) [[1](#_ENREF_17)7] | Portugal (Lisbon and Madeira) | 54.6±2.3  7.4±2.9 | 54  128 | – | Exposed to solid waste incinerator  Not exposed |
| Ettinger et al. (2007) [[18](#_ENREF_18)] | Mexico | Ca supplementation: 3.8±2.0^b^  No Ca supplementation: 4.1±2.0^b^ | 334  336 | 2001–2003 |  |
| Wang et al. (2008) [[1](#_ENREF_19)9] | China (Shanghai) | 6.43^a^ | 130 | 2006–2007 | – |
| Al-Jawadi et al. (2009) [[2](#_ENREF_20)0] | Iraq (Mosul) | 3.26±1.9 ^b^ | 350 | – | Leaded petrol in use |
| Rudge et al. 2009 | South Africa | 2.3 (IQR 1.46–3.10) | 62 | – |  |
| Mirghani (2010) [[2](#_ENREF_21)1] | Saudi Arabia (Aseer region) | <20 : 70.1%  >20: 29.9% | 94  40 | – | Holiday resort area thought to be free of lead as air pollution from oil processing |
| Amaral et al. 2010 [[22](#_ENREF_22)] | Brazil (San Paulo) | 1.736±0.009 | 120 | – |  |
| Lin et al. 2010 [[23](#_ENREF_23)] | Taiwan | 1.71±1.11 | 308 | 2004–2005 |  |
| Al Saleh et al. 2011 [[24](#_ENREF_24)] | Saudi Arabia (nr Riyadh) | 2.897±1.851 | 1577 | – |  |
| Vigeh et al. 2011 [[25](#_ENREF_25)] | Iran (Tehran) | 3.72±2.03  4.52±1.63 | 304  44 | – | Not preterm  Preterm |
| Njoku and Orisakwe (2012) [[2](#_ENREF_26)6] | Nigeria (Imo State) | 99±123 | 90 | – | Contaminated farmland |
| Ugwuja et al. (2012) [[27](#_ENREF_27)] | Nigeria (Ebony State) | 36.4±18.5 (2.7–73.8) | 349 | – |  |
| Tiwari et al. (2012) [[28](#_ENREF_28)] | India (Lucknow) | 1.84±0.12  1.98±0.13  2.61±0.11  3.62±0.17 | 50  50  50  25 | – | Control  Mild anaemia  Moderate anaemia  Severe anaemia |
| Motawei et al. (2013) [[29](#_ENREF_29)] | Egypt (Dakhalia) | 37.7±9.2  14.5±3.2 | 115  25 | – | Pre-eclampsia  No pre-eclampsia |

Studies shown are those published from 2000 onwards only.

^a^Median; ^b^geometric mean; values in parentheses are ranges.

**References**

1. Bjerregaard P, Hansen JC (2000) Organochlorines and heavy metals in pregnant women from the Disko Bay area in Greenland. Sci Total Environ 245: 195-202.

2. Raghunath R, Tripathi RM, Sastry VN, Krishnamurthy TM (2000) Heavy metals in maternal and cord blood. Science of the Total Environment 250: 135-141.

3. Durska G (2001) [Levels of lead and cadmium in pregnant women and newborns and evaluation of their impact on child development]. Ann Acad Med Stetin 47: 49-60.

4. Ataniyazova OA, Baumann RA, Liem AK, Mukhopadhyay UA, Vogelaar EF, et al. (2001) Levels of certain metals, organochlorine pesticides and dioxins in cord blood, maternal blood, human milk and some commonly used nutrients in the surroundings of the Aral Sea (Karakalpakstan, Republic of Uzbekistan). Acta Paediatr 90: 801-808.

5. Sanin LH, Gonzalez-Cossio T, Romieu I, Peterson KE, Ruiz S, et al. (2001) Effect of maternal lead burden on infant weight and weight gain at one month of age among breastfed infants. Pediatrics 107: 1016-1023.

6. Smargiassi A, Takser L, Masse A, Sergerie M, Mergler D, et al. (2002) A comparative study of manganese and lead levels in human umbilical cords and maternal blood from two urban centers exposed to different gasoline additives. Sci Total Environ 290: 157-164.

7. Hanning RM, Sandhu R, MacMillan A, Moss L, Tsuji LJ, et al. (2003) Impact on blood Pb levels of maternal and early infant feeding practices of First Nation Cree in the Mushkegowuk Territory of northern Ontario, Canada. J Environ Monit 5: 241-245.

8. Yao HY, Huang XH (2003) The blood lead level and pregnant outcome in pregnant women with non-occupational lead exposure. Chinese Journal of Obstetrics and Gynaecology 38: 340-342.

9. Rahman A, Hakeem A (2003) Blood lead levels during pregnancy and pregnancy outcome in Karachi women. J Pak Med Assoc 53: 529-533.

10. Wang C, Huang L, Zhou X, Xu G, Shi Q (2004) Blood lead levels of both mothers and their newborn infants in the middle part of China. Int J Hyg Environ Health 207: 431-436.

11. Kirel B, Aksit MA, Bulut H (2005) Blood lead levels of maternal-cord pairs, children and adults who live in a central urban area in Turkey. Turk J Pediatr 47: 125-131.

12. Butler Walker J, Houseman J, Seddon L, McMullan E, Tofflemire K, et al. (2006) Maternal and umbikical cord blood levels of mercury, lead, cadmium, and essential trace elements in Arctic Canada. Enviromental Research 100: 295-319.

13. Vigeh M, Yokoyama K, Ramezanzadeh F, Dahaghin M, Sakai T, et al. (2006) Lead and other trace metals in preeclampsia: a case-control study in Tehran, Iran. Environ Res 100: 268-275.

14. Kawata K, Li Y, Liu H, Zhang XQ, Ushijima H (2006) Specific factors for prenatal lead exposure in the border area of China. Int J Hyg Environ Health 209: 377-383.

15. Lamadrid-Figueroa H, Tellez-Rojo MM, Hernandez-Cadena L, Mercado-Garcia A, Smith D, et al. (2006) Biological markers of fetal lead exposure at each stage of pregnancy. J Toxicol Environ Health A 69: 1781-1796.

16. Eik Anda E, Nieboer E, Dudarev AA, Sandanger TM, Odland JO (2007) Intra- and intercompartmental associations between levels of organochlorines in maternal plasma, cord plasma and breast milk, and lead and cadmium in whole blood, for indigenous peoples of Chukotka, Russia. J Environ Monit 9: 884-893.

17. Reis MF, Sampaio C, Brantes A, Aniceto P, Melim M, et al. (2007) Human exposure to heavy metals in the vicinity of Portuguese solid waste incinerators--Part 2: biomonitoring of lead in maternal and umbilical cord blood. Int J Hyg Environ Health 210: 447-454.

18. Ettinger AS, Hu H, Hernandez-Avila M (2007) Dietary calcium supplementation to lower blood lead levels in pregnancy and lactation. J Nutr Biochem 18: 172-178.

19. Wang P, Tian Y, Shi R, Zou XY, Gao Y, et al. (2008) Study on maternal-fetal status of Pb, As, Cd, Mn and Zn elements and the influence factors. Chinese Journal of Preventive Medicine 42: 722-726.

20. Al-Jawadi AA, Al-Mola ZW, Al-Jomard RA (2009) Determinants of maternal and umbilical blood lead levels: a cross-sectional study, Mosul, Iraq. BMC Res Notes 2: 47.

21. Mirghani Z (2010) Effect of low lead exposure on gestational age, birth weight and premature rupture of the membrane. J Pak Med Assoc 60: 1027-1030.

22. Amaral JH, Rezende VB, Quintana SM, Gerlach RF, Barbosa F, Jr., et al. (2010) The relationship between blood and serum lead levels in peripartum women and their respective umbilical cords. Basic Clin Pharmacol Toxicol 107: 971-975.

23. Lin CM, Doyle P, Wang DL, Hwang YH, Chen PC (2010) The role of essential metals in the placental transfer of lead from mother to child. Reproductive Toxicology 29: 443-446.

24. Al-Saleh I, Shinwari N, Mashhour A, Mohamed Gel D, Rabah A (2011) Heavy metals (lead, cadmium and mercury) in maternal, cord blood and placenta of healthy women. Int J Hyg Environ Health 214: 79-101.

25. Vigeh M, Yokoyama K, Seyedaghamiri Z, Shinohara A, Matsukawa T, et al. (2011) Blood lead at currently acceptable levels may cause preterm labour. Occup Environ Med 68: 231-234.

26. Njoku CO, Orisakwe OE (2012) Higher blood lead levels in rural than urban pregnant women in Eastern Nigeria. Occupational and Environmental Medicine 69: 850-851.

27. Ugwuja EI, Ibiam UA, Ejikeme BN, Obuna JA, Agbafor KN (2012) Blood Pb Levels in pregnant Nigerian women in Abakaliki, South-Eastern Nigeria. Environ Monit Assess.

28. Tiwari AKM, Mahdi AA, Zahra F, Sharma S, Negi MPS (2012) Evaluation of low blood lead levels and its association with oxidative stress in pregnant anemic women: a comparative prospective study. Ind J Clin Biochem 27: 246-252.

29. Motawei SM, Attalla SM, Gouda HE, El-Harouny MA, El-Mansoury AM (2013) Lead levels in pregnant women suffering for pre-eclamsia in Dakahlia, Egypt. Int J Occup Environ Med 4: 36-44.
